# Behavioral and Immune Responses to Infection Require Gαq- RhoA Signaling in *C. elegans*

**DOI:** 10.1371/journal.ppat.1002530

**Published:** 2012-02-16

**Authors:** Rachel McMullan, Alexandra Anderson, Stephen Nurrish

**Affiliations:** 1 MRC Cell Biology Unit, MRC Laboratory for Molecular Cell Biology and Department of Neuroscience, Physiology and Pharmacology, University College London, London, United Kingdom; 2 Division of Cell and Molecular Biology, Department of Life Sciences, Imperial College London, South Kensington Campus, London, United Kingdom; Harvard Medical School, United States of America

## Abstract

Following pathogen infection the hosts' nervous and immune systems react with coordinated responses to the danger. A key question is how the neuronal and immune responses to pathogens are coordinated, are there common signaling pathways used by both responses? Using *C. elegans* we show that infection by pathogenic strains of *M. nematophilum,* but not exposure to avirulent strains, triggers behavioral and immune responses both of which require a conserved Gαq-RhoGEF Trio-Rho signaling pathway. Upon infection signaling by the Gαq pathway within cholinergic motorneurons is necessary and sufficient to increase release of the neurotransmitter acetylcholine and increase locomotion rates and these behavioral changes result in *C. elegans* leaving lawns of *M. nematophilum*. In the immune response to infection signaling by the Gαq pathway within rectal epithelial cells is necessary and sufficient to cause changes in cell morphology resulting in tail swelling that limits the infection. These Gαq mediated behavioral and immune responses to infection are separate, act in a cell autonomous fashion and activation of this pathway in the appropriate cells can trigger these responses in the absence of infection. Within the rectal epithelium the Gαq signaling pathway cooperates with a Ras signaling pathway to activate a Raf-ERK-MAPK pathway to trigger the cell morphology changes, whereas in motorneurons Gαq signaling triggers behavioral responses independent of Ras signaling. Thus, a conserved Gαq pathway cooperates with cell specific factors in the nervous and immune systems to produce appropriate responses to pathogen. Thus, our data suggests that ligands for Gq coupled receptors are likely to be part of the signals generated in response to *M. nematophilum* infection.

## Author Summary

Once infected by a pathogen the nervous and immune systems of many animals react with coordinated responses to the danger. A key question is what are the pathways by which responses to infection occur and to what extent are the same pathways involved in differing responses? Here we demonstrate that a Gαq-RhoA pathway is required for both behavioral and immune responses to infection in *C. elegans*. We show that Gαq-RhoA signaling is a late step in the response to infection and their site of action defines the cellular targets of signals generated internally in response to infection. One response is to move away from sites of pathogenic bacteria and Gαq-RhoA signaling acts in motorneurons to achieve this. A second response is an innate immune response where Gαq-RhoA signaling acts within cells close to sites of infection, the rectal epithelial cells, to cause major changes in their size and shape to mitigate the effects of infection. Our work demonstrates that ligands for Gq coupled GPCRs are likely to be required for response to infection. Identifying these ligands and the cells that release them will help define the mechanisms by which *C. elegans* recognizes pathogens and coordinates behavioral and immune responses to infection.

## Introduction

Animals have evolved multiple strategies for coping with the presence of pathogenic microbes. The best characterized is the immune response where animals activate their physical and cellular defenses to respond to invading microorganisms. The innate immune response is the first line of this defense, acting to recognize and eliminate pathogens [1], [2], [3]. Unlike adaptive immunity; which is only found in vertebrates, innate immunity is highly conserved throughout evolution with plants, invertebrates and vertebrates sharing surprisingly similar responses including expression of antimicrobial peptides and activation of phagocytosis. As a consequence of this, invertebrate model systems, including *Drosophila* and *Caenorhabditis elegans,* have provided important insights into the molecular mechanisms that underlie infection responses [4], [5], [6], [7] *C. elegans* is able mount innate immune responses to both naturally occurring (*Nematocida parisii, Drechmeria coniospora* and *Microbacterium nematophilum*) and clinically important (*Pseudomonas aeruginosa* and *Staphylococcus aureus*) bacterial and fungal pathogens when they are provided as a food source [7], [8], [9], [10], [11], [12], [13]. Because it lacks professional immune cells and phagocytes *C. elegans* relies on epithelial innate immunity to mount a response that includes transcription of many host defense genes [14] including numerous anti-microbial peptides [15]. It is becoming increasingly clear that this type of epithelial immunity also plays an important role in the immune response of the mammalian intestine [16].

Changes in neuronal signaling also occur upon infection and neuronal signaling can modulate the innate immune response [17]. In addition, behavioral changes can also be triggered by exposure to pathogen. For example, avoidance of pathogens is likely to be an important part of the response to microbes in many animals and perhaps even humans [18]. Studies of pathogen avoidance have utilized *C. elegans*, which has evolved rapid avoidance behaviors allowing it to alter its locomotion in response to aversive cues in its environment [19], [20], [21], [22]. Aversive cues such as serrawettin, a secreted surfactant produced by *Serratia marcescens*, are directly sensed by chemosensory neurons located in the animal's head [19]. The receptors for these pathogen-associated cues are unknown, however, the G-protein ODR-3 and the TAX-2/4 cGMP gated channel are required to mediate avoidance to *S. marcescens* [19] and TAX-2/4 is also required for animals to avoid *M. nematophilum* and *P. aeruginosa* [23] implicating G-protein coupled receptors (GPCRs) in at least some of these responses. A conserved MAP Kinase pathway including p38 MAPK has also been shown to regulate both the innate immune response and aversive behavior to *Pseudomonas aeruginosa* [24] and neuronal TGF-ß signaling is important for the induction of antimicrobial peptides upon infection by *D. coniospora* [25]. Some of these behavioral responses are likely to involve detection of pathogen by chemosensory neurons, for example, serotonin release from ADF chemosensory neurons is required for learnt aversive responses to pathogenic bacteria [22], dopamine release from sensory neurons is required for behavioral responses to enteropathic *E. coli* [26], and a p38 MAPK pathway is required in chemosensory neurons to mediate changes in egg laying in response to *P. aeruginosa* [24].

Behavioral changes such as aversion require changes in locomotion. Within *C. elegans* cholinergic motor neurons Gαq (EGL-30), Gα12 (GPA-12) and Gαo (GOA-1) comprise a G-protein coupled regulatory network that controls the release of acetylcholine (ACh) at the neuromuscular junction [27] by regulating diacylglycerol (DAG) levels at the synapse [28]. EGL-30 (Gαq) is central to this regulatory network and mediates DAG production through regulation of EGL-8 (PLCß) [29]. DAG produced by EGL-8 (PLCß) is also required for activation of the PKC homolog TPA-1 in the response to infection by the fungus *D. coniospora* [30]. However, this role for DAG in response to infection does not involve neurons. More recently Gαq (EGL-30) has also been shown to regulate DAG destruction by directly activating the Trio ortholog UNC-73 (RhoGEF) resulting in activation of the small GTPase RHO-1 (the single *C. elegans* Rho ortholog), which negatively regulates the diacylglycerol kinase DGK-1 [31], [32]. Reduction-of-function mutations in EGL-30 (Gαq) are lethargic and gain-of-function mutants have hyperactive locomotion [33]. Animals with mutations in UNC-73 (Trio) also move lethargically [32], [34]. Similarly, inhibiting endogenous RHO-1 signaling by expressing the Rho inhibitor, C3 transferase, in the cholinergic motor neurons leads to lethargic locomotion and a decrease in ACh release [31]. Thus, changes in Gαq-RhoGEF Trio-Rho signaling result in changes in ACh release and locomotion rate.

Although a great deal has been discovered about the G-protein pathways that control neuronal activity in the cholinergic motor neurons less well understood are the signals that act upon the GPCRs to regulate G-protein signaling. Almost certainly changes in the environment will alter activity of the cholinergic motor neurons and thus locomotion. In its natural environment *C. elegans* is constantly sensing and responding to attractive and aversive signals by altering its locomotion and animals that have evolved effective mechanisms for interpreting and responding to environmental cues, such as the presence of pathogen, will have an evolutionary advantage. A recent study has shown that EGL-30 (Gαq) signaling in the chemosensory neuron, ASH, is required for the response to some aversive stimuli [35]. Is the Gαq-RhoGEF Trio-Rho pathway part of the signaling network that modulates neuronal activity and alters locomotion in response to the presence of pathogen, and if so in which cells is this pathway required? In order to understand more about how the regulation of Gαq signaling modulates neuronal activity in response to pathogens we have investigated the role of EGL-30 (Gαq) in the response to infection by the nematode-specific pathogen *M. nematophilum*. *M. nematophilum* colonizes the rectum of *C. elegans* causing it to mount an innate immune response that includes the induction of several antimicrobial factors, swelling of the tail and an aversive behavior that causes animals to leave lawns of *M. nematophilum* [9], [23], [36].

Here we show that upon infection by *M. nematophilum* pathogen *C. elegans* alters locomotion behavior: we observe an increase in both ACh release and locomotion in response to infection that requires the Gαq-Rho GEF Trio-Rho signaling pathway in the cholinergic motorneurons and that this signaling is required for aversive behavior. We also show that the innate immune response to *M. nematophilum* infection requires the Gαq-Rho GEF Trio-Rho signaling pathway. Activation of this pathway in neurons is sufficient to trigger the behavioral response to pathogen, but in epithelial cells it must co-operate with a Ras signaling pathway to trigger the innate immune response. Thus, our studies demonstrate that the Gαq-Rho GEF Trio-Rho signaling pathway is a core pathway acting either alone or in combination with other pathways in a cell specific manner to trigger behavioral and innate immune responses to pathogen.

## Results

### Gαq signaling mediates behavioral responses to infection

**Figure 1:**
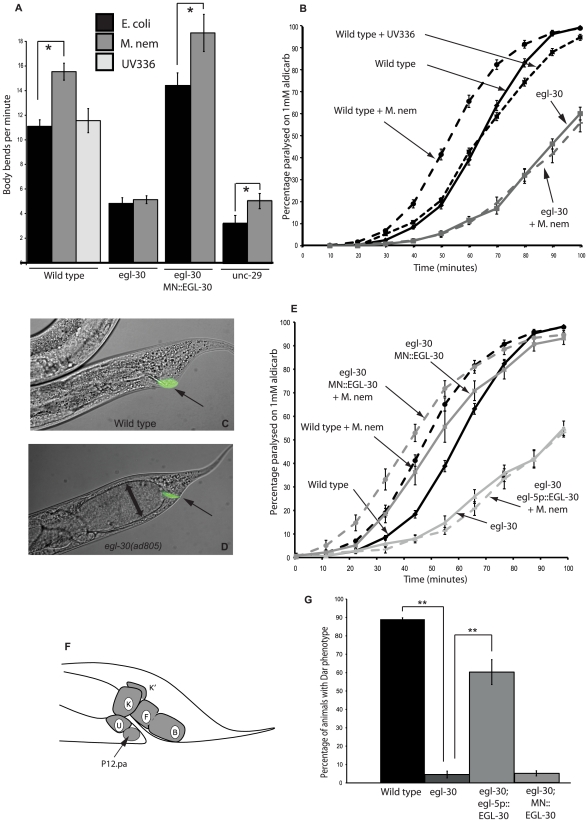
EGL-30 (Gαq) signaling is required in different tissues for behavioral and immune responses to infection. The locomotion rate of wild type and *unc-29(e1072)* animals was increased following infection with *M. nematophilum* (A). No increase was observed in *egl-30(ad805)* loss-of-function mutants (A). Synaptic release of endogenous acetylcholine was measured by determining the onset of paralysis induced by the acetylcholine esterase inhibitor aldicarb. Infection of wild type animals with *M. nematophilum* resulted in a faster onset of aldicarb-induced paralysis relative to wild type controls grown on *E. coli*, suggesting an increase in the levels of ACh release following infection (B). In contrast *egl-30(ad805)* was resistant to aldicarb and infection of these animals did not increase ACh release (B). Mutations in *egl-30(ad805)* significantly decreased the percentage of Dar animals observed following *M. nematophilum* infection although bacteria, labeled in green using the nucleic acid stain SYTO13, still attached to the anal opening (C, D and G) (rectal opening is indicated with an arrow in C and D). These animals were severely constipated and the intestinal distention is indicated by a double-headed arrow (D). Expression of EGL-30 (Gαq) in the rectal epithelium (F kindly drawn by H. Chamberlin) using a 1.3 Kb fragment of the *egl-5* promoter (*egl-5p*::EGL-30; *egl-30(ad805)*) was sufficient to rescue the Dar response following infection (G) however these animals remained resistant to aldicarb and ACh release was not increased following infection (E). In contrast cholinergic motorneuron expression of EGL-30 (Gαq) from the *unc-17* promoter (MN::EGL-30) rescued increases in locomotion (A) and ACh release following infection (E) but not the Dar response (G). P values between 0.05 and 0.001 (*), and P values of 0.001 or less (**).

We, and others, have previously characterized an extensive network of G-protein signaling pathways that regulate ACh release and locomotion in the cholinergic motor neurons of *C. elegans* [28]. An important question is what are the environmental inputs into this network of neuronal signaling pathways that trigger changes in the activity of the cholinergic motor neurons? One important environmental cue would be the presence of pathogens; it would be an advantage, upon infection, for animals to alter their locomotion to move away from the location of the pathogen and this has been demonstrated in a number of cases [37]. This proved to be correct as wild-type *C. elegans* increased their rate of locomotion upon exposure to the pathogen *M. nematophilum* relative to animals grown on control OP50 *E. coli* (Figure 1A). Mutations in *C. elegans* EGL-30 (Gαq) (*egl-30(ad805))* caused a decrease in locomotion and these mutants did not change their locomotion in response to exposure to *M. nematophilum* indicating that signaling via EGL-30 (Gαq) is required to alter locomotion behavior in response to exposure to *M. nematophilum* (Figure 1A). It is possible that the reduced locomotion of *egl-30(ad805)* animals makes it impossible for us to detect small increases in locomotion caused by exposure to *M. nematophilum.* Mutations in the UNC-29 nicotinic ACh receptor (*unc-29(e1072)*) [38] cause a stronger reduction in locomotion than *egl-30(ad805)*, however, these mutants still increased rates of locomotion in response to exposure to *M. nematophilum*, confirming that we can detect differences in locomotion rate caused by exposure to *M. nematophilum* in mutants that move slowly. Increased locomotion of *C. elegans* relative to controls could represent a specific response to exposure to a pathogen or a non-specific difference between *M. nematophilum* and our control bacteria (the OP50 *E. coli* strain) as a food source, for example animals growing on *M. nematophilum* could be starved relative to animals growing on *E. coli.* To determine which explanation is likely to be correct we exposed *C. elegans* to the UV336 *M. nematophilum* strain, which is unable to infect *C. elegans* [39]. Animals grown on UV336 did not change their locomotion compared to controls suggesting that wild-type animals increased their rate of locomotion upon infection by *M. nematophilum* (Figure 1A). We have previously shown that EGL-30 (Gαq) acts within the cholinergic motorneurons to regulate locomotion. Expression of EGL-30 from the *unc-17* cholinergic motorneuron specific promoter (MN::EGL-30) not only restored the locomotion of EGL-30 (Gαq) mutant animals it also caused the animals to move faster than wildtype animals. Expression of EGL-30 (Gαq) in just the cholinergic motorneurons also restored the increased locomotion response of animals in response to infection by *M. nematophilum* compared to *E. coli* (Figure 1A).

We next examined the effect of infection on ACh release at the *C. elegans* neuromuscular junction using the acetylcholine esterase inhibitor aldicarb. Aldicarb prevents the removal of endogenously released ACh causing it to build up and resulting in hyper-contraction of the body wall muscles that paralyses the animal with a time course dependent on the rates of release from the cholinergic motor neurons [40]. Animals with decreased levels of ACh release are resistant to aldicarb-induced paralysis [41]. Exposure of wild type animals to *M. nematophilum* resulted in an increase in ACh release as shown by hypersensitivity to aldicarb compared to animals grown on *E. coli* (Figure 1B). Exposure to the avirulent UV336 *M. nematophilum* strain did not alter levels of ACh release suggesting that changes in ACh release are in response to infection by *M. nematophilum.* Reduction-of-function mutations in EGL-30 (Gαq) are resistant to aldicarb ([29] and Figure 1B) and infection of *egl-30(ad805)* did not result in an increase in ACh release (Figure 1B) indicating that EGL-30 (Gαq) signaling is required to increase ACh release and alter locomotion behavior in response to infection. Expression of EGL-30 (Gαq) only within the cholinergic motorneurons (MN::EGL-30) rescued the decreased ACh release defect in *egl-30(ad805)* mutant animals and caused a level of ACh release higher than that of wild-type animals. Cholinergic expression of EGL-30 (Gαq) was also sufficient to restore the increased levels of ACh release in response to infection by *M. nematophilum* compared to *E. coli* (Figure 1E). Thus, our results are consistent with a role for EGL-30 (Gαq) within the cholinergic motorneurons that is necessary and sufficient to mediate the increased locomotion response and the increased ACh release response of *C. elegans* infection by *M. nematophilum*.

### Gαq signaling mediates immune responses to infection

Upon *M. nematophilum* infection of wild type *C. elegans* the pathogen adheres to the cuticle around the rectal opening causing the animal to mount an innate immune response that includes swelling around this opening known as the Deformed anal region (Dar) phenotype [9] (Figure 1C and G). While carrying out our locomotion assays we noticed that the Dar phenotype was significantly decreased in *egl-30(ad805)* animals following infection. The *egl-30(ad805)* mutation did not alter the ability of the pathogen to attach to the cuticle, as Syto13-labelled *M. nematophilum* was still observed adhering to the rectum (Figure 1D and G). In addition to tail swelling, infection with *M. nematophilum* causes constipation [9]. This is exacerbated in animals that are defective in the Dar response [42]. Consistent with their decreased Dar response *egl-30(ad805)* animals became severely constipated following infection, but not when grown on *E. coli* (Figures 1D and S1). Thus, EGL-30 (Gαq) signaling is required for both behavioral and innate immune responses to infection.

Expression of EGL-30 (Gαq) within the cholinergic motorneurons was unable to rescue the Dar response of *egl-30(ad805)* mutants (Figure 1G). Expression of EGL-30 (Gαq) cDNA from a 1.3 Kb *egl-5* promoter fragment that is expressed in the B, K, F, U, and P12.pa rectal epithelial cells and in three posterior body wall muscles [43] (Figure 1F) did rescue the Dar phenotype in *egl-30(ad805)* animals, however, these animals remained constipated (Figures 1G and S1). *egl-30(ad805)* animals expressing EGL-30 (Gαq) in the rectal epithelial cells remained resistant to aldicarb and no increase in ACh release was observed following infection (Figure 1E). Our data suggest the EGL-30 (Gαq) regulates behavioral responses to infection by *M. nematophilum* by acting in the cholinergic motorneurons and innate immune responses to infection by acting in the rectal epithelial cells.

Expression of constitutively active EGL-30(Q205L) in cholinergic motorneurons or from a heat shock-inducible promoter is sufficient to increase both locomotion and ACh release [29]. To determine whether EGL-30 (Gαq) signaling was sufficient to induce the Dar response in the absence of infection we generated transgenic animals that expressed constitutively active EGL-30(Q205L) in adult animals (using a heat shock-inducible promoter) or in the rectal epithelial cells (using a 1.3 Kb fragment of the *egl-5* promoter). Over expression of activated EGL-30 (Gαq) from these transgenes resulted in tail swelling in the absence of infection (Table 1 and data not shown) suggesting that EGL-30 (Gαq) signalling in the adult rectal epithelial cells is sufficient to cause the Dar phenotype. A gain-of-function mutation in the chromosomal *egl-30* (*egl-30(js126)*) gene has also been isolated [44]. In contrast to transgenic expression of activated *egl-30* this chromosomal mutation did not trigger the Dar response (Table 1). The inability of the *egl-30(js126)* mutation to activate an innate immune response is in contrast to cholinergic motor neurons where this mutation is sufficient to increase locomotion and ACh release [29], [32].

**Table 1:** Analysis of genetic interactions between Rho, Ras and Gαq signaling in the immune response.

Genotype	% animals with *dar* phenotype ± s.e.m	n value
hs::EGL-30*+heat shock	68.5±3.2	6
hs::EGL-30*;*unc-73(ce362)*+heat shock	5.6±1.7^a^	5
hs::EGL-30*;*egl-8(md1971)*+heat shock	2.6±1.4^a^	3
hs::EGL-30*;*let-60(n1046gf)*+heat shock	81.7±4.2^b^	4
*let-60(n1046gf)*	0±0	7
*egl-30(js126gf)*	0±0	7
*egl-30(js126gf);let-60(n1046gf)*	8.9±3.5^c^	7

The number of dar animals was scored as a percentage of the total. Heat shock was as described in the Material and Methods. Values are means +/− the standard error.

a = p<0.001 relative to hs::EGL-30*+heat shock alone.

b = p<0.05 relative to hs::EGL-30*+heat shock alone.

c = p<0.05 relative to single mutants.

### The RhoGEF UNC-73(Trio) is required for the *C. elegans* immune and behavioural responses to infection

EGL-30 (Gαq) signaling in the cholinergic motor neurons activates at least two pathways to regulate ACh release [45]. Firstly EGL-30 (Gαq) activates the PLCß, EGL-8, to increase diacylglycerol (DAG) production [29] and secondly it binds to and activates the RhoGEF UNC-73 (Trio) [32] to regulate signaling via RHO-1 and decrease DAG destruction [45]. Mutations in EGL-8 (PLCß) and UNC-73(Trio) suppress the increased locomotion and ACh release caused by activation of EGL-30 (Gαq) [29], [32]. Does EGL-30 (Gαq) utilise the same pathways during the Dar response to infection? To determine whether UNC-73 (Trio) and EGL-8 (PLCß) are also required downstream of EGL-30 (Gαq) during the immune response we induced the Dar phenotype in the absence of infection using a heat shock-inducible gain-of-function EGL-30(Q205L). Following heat shock the Dar phenotype observed in these animals was suppressed by *egl-8(md1971)* and *unc-73(ce362)* mutants (Table 1) placing PLß and Rho signaling downstream of EGL-30 (Gαq) in the immune response to infection. Consistent with our results mutations in EGL-8 (PLCß) were identified in a screen for suppressors of the infection-induced Dar phenotype [23] suggesting that conserved signaling pathways may act in multiple tissues to regulate different responses to infection. Here we investigate the role of UNC-73 (Trio) and its effector RHO-1 in the response to *M. nematophilum* infection.

**Figure 2:**
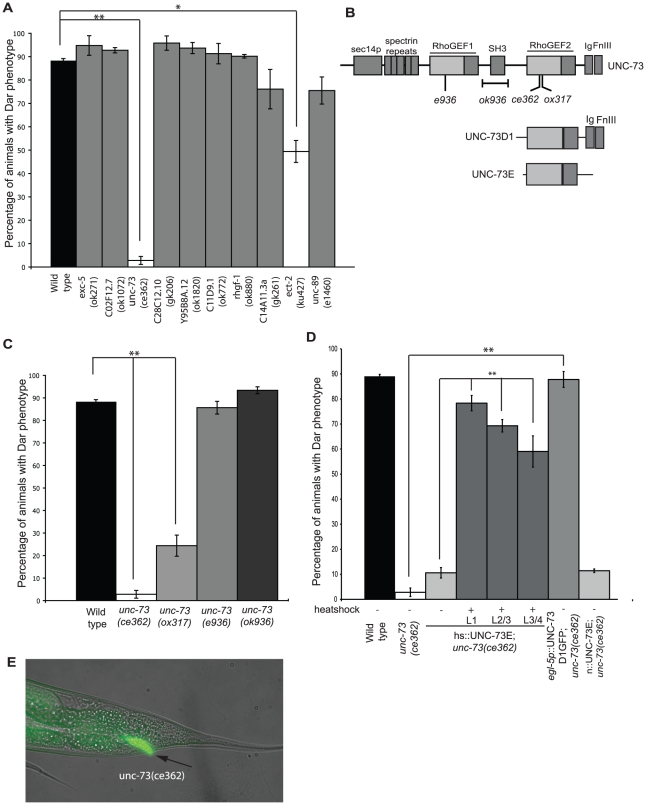
UNC-73 (Trio) is required in rectal epithelial cells for the Dar response to infection. Viable RhoGEF mutants were infected with *M. nematophilum* and the percentage of Dar animals scored. Mutations in *unc-73(ce362)*, and *ect-2(ku427)*, but not other RhoGEF's, significantly decreased the percentage of Dar animals (A). The UNC-73 gene contains two RhoGEF domains, one specific for Rac (RhoGEF1) and the other specific for Rho (RhoGEF2) (B). Animals with mutations that prevented Rac activation (*unc-73(e936)* and *(ok936)*) had a wild-type Dar response whereas mutations in RhoGEF2 (*unc-73(ce362)* and *(ox317)*) significantly decreased the percentage of Dar animals (C). Expression of UNC-73 isoforms E or D1 using heat shock at L1, L2/L3 and L3/L4 stage (hs::UNC-73E) or rectal epithelial (*egl-5p*::UNC-73D1::GFP), but not neuronal (n::UNC-73E), expression rescued the Dar phenotype in *unc-73(ce362)* animals (D). Although *unc-73(ce362)* animals failed to produce a Dar response *M. nematophilum* bacteria, labeled using the nucleic acid stain SYTO13, still attached to the anal opening (E), the rectal opening is indicated with an arrow).

The *C. elegans* genome encodes 21 Dbl containing Rho GEF's several of which are required for viability [46], [47]. To investigate whether UNC-73 (Trio) was the only Rho GEF required for the innate immune response we infected viable, fertile animals carrying mutations in 10 of the 21 known *C. elegans* Rho GEF's with *M. nematophilum*. Following infection only *unc-73(ce362)* and *ect-2(ku427)* significantly decreased the percentage of infected animals with a Dar phenotype, indicating that a subset of Rho signaling pathways are required for the pathogen-induced Dar response (Figure 2A). UNC-73 (Trio) is a highly conserved RhoGEF related to mammalian Trio [34]. It contains two tandem RhoGEF domains: the N-terminal RHOGEF1 domain specifically activates Rac family GTPases, whereas the C-terminal RHOGEF2 domain specifically activates RHO-1 [48] (Figure 2B). Mutations that selectively disrupted *unc-73*′s RacGEF activity (*e936* and *ok936*) [34] had a normal pathogen-induced Dar response, whereas mutations specific to the RhoGEF domain (*ce362 and ok317*) [32] had a decreased response (Figure 2C) although pathogen was still able to attach to the cuticle, as Syto13-labelled *M. nematophilum* was observed adhering to the rectum of *unc-73(ce362)* animals (Figure 2E). Furthermore, the pathogen-induced Dar could be rescued in *unc-73(ce362)* mutants by expressing UNC-73 (Trio) isoforms that only contain the RHOGEF2 domain [34] (Figure 2B and D), confirming that RHO-1, but not Rac, activation by UNC-73 (Trio) is required for the Dar response to pathogen. Henceforth all the UNC-73 (Trio) mutations used are in the RHOGEF2 domain that selectively blocks RHO-1 activation.

**Figure 3:**
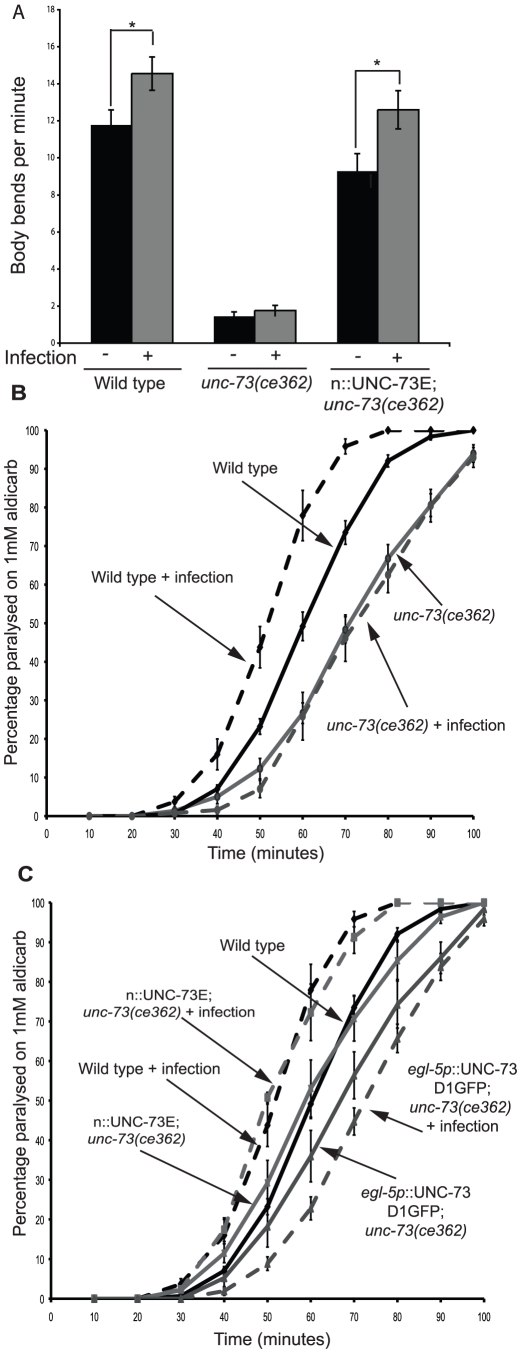
UNC-73 (Trio) is required in neurons for the behavioral response to infection. Animals carrying a mutation in *unc-73(ce362)* did not significantly change their locomotion rate when infected by *M. nematophilum* and this effect was rescued by expression of UNC-73E from a pan-neuronal promoter (n::UNC-73E) (A). *unc-73(ce362)* mutants were slightly resistant to aldicarb when grown on *E. coli* (B). ACh release was not increased in these animals following infection (B). Expression of UNC-73 in neurons (n::UNC-73E), but not in the rectal epithelial cells (*egl-5p*::UNC-73D1GFP), was sufficient to rescue the increase in ACh release upon infection by *M. nematophilum* of *unc-73(ce362)* mutants (C). P values between 0.05 and 0.001 (*), and P values of 0.001 or less (**).

Because UNC-73 (Trio) was required for the Dar phenotype and has previously been shown to regulate *C. elegans* locomotion under standard conditions [32] we next asked whether Rho signaling was also required to alter locomotion behavior and increase ACh release following infection. Unlike wild type controls, *unc-73(ce362)* animals did not increase their locomotion rate following infection (Figure 3A). Expression of UNC-73E from a pan-neuronal promoter partially rescued the reduced locomotion phenotype and restored the increase in locomotion following infection. *unc-73(ce362)* animals were slightly resistant to aldicarb when grown on *E. coli* OP50 as has been observed previously [34] and ACh release was not increased following infection (Figure 3B) indicating that UNC-73(Trio) is required for both the immune and behavioral responses to infection.

Rho signaling is required throughout development [49]. Therefore to investigate whether UNC-73 was required in adult animals for the Dar phenotype we performed rescue experiments in *unc-73(ce362)* animals using a heat shock-inducible UNC-73 transgene. We were able to partially rescue the Dar phenotype in *unc-73(ce362)* adults by expressing UNC-73 10–18 hours prior to adulthood (L3/L4 larval stage) indicating that Rho signaling in adult animals is required for the response (Figure 2D).

To determine the site of action for UNC-73 (Trio) in both the behavioral and immune responses to infection we performed rescue experiments using UNC-73 expressed from either the neuronal specific promoter *rab-3* or in the rectal epithelial cells using a 1.3 Kb *egl-5* promoter fragment. Expression of UNC-73 in the rectal epithelial cells was sufficient to rescue the defective Dar response of *unc-73(ce362)* mutants (Figure 2D) however these animals remained resistant to aldicarb and no increase in neurotransmitter release was observed following infection (Figure 3C). Conversely expression of UNC-73 in the nervous system failed to rescue the Dar response (Figure 2D) but wild type levels of neurotransmitter release were observed in these animals in the absence of infection (Figure 3C). *M. nematophilum* infection of these animals resulted in an increase in ACh release that was identical to the one observed following infection of wild type animals (Figure 3C). Taken together this data confirms that the Gαq-RhoGEF Trio signaling pathway acts in different tissues to mediate the behavioral and immune responses to infection.

### Activation of Rho signaling in the *C. elegans* adult rectal epithelial cells alters cell morphology and mimics the innate immune response to infection

**Figure 4:**
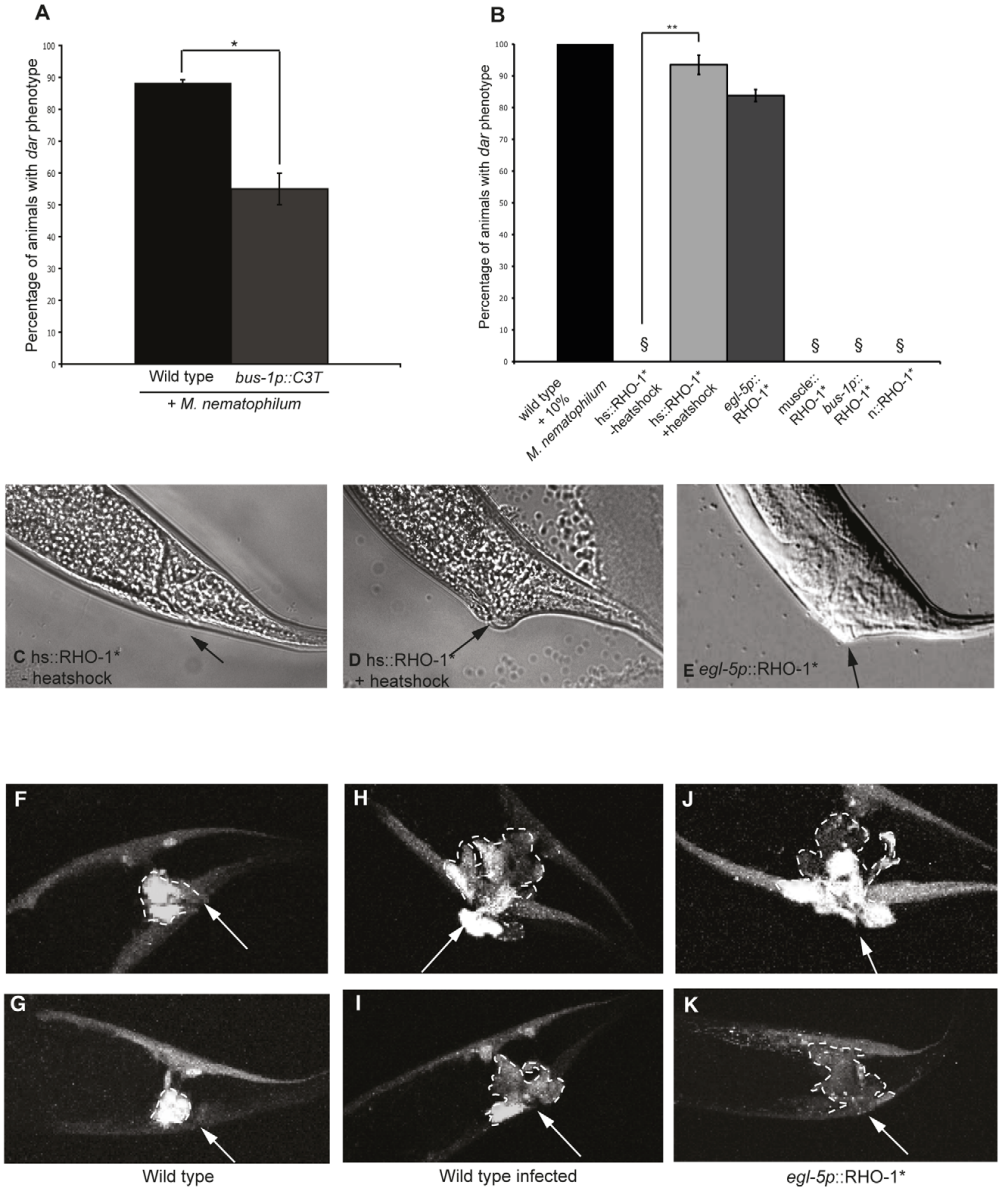
Rho signaling in the adult rectal epithelium causes tail swelling and changes cell morphology. Inhibition of Rho in a subset of rectal epithelial cells using the Rho inhibitor C3 Transferase expressed from the *bus-1* promoter (*bus-1p*::C3T) significantly decreased the percentage of Dar animals (A). Expression of RHO-1* in adult animals using a heat shock-inducible promoter triggers the Dar response (B and D), in the absence of heat shock animals expressing hs::RHO-1* were wild type (B and C). Cell specific expression of RHO-1* in the rectal epithelial cells (*egl-5p*::RHO-1*); but not in neurons (n::RHO-1*) or muscle (muscle::RHO-1*), also resulted in tail swelling (B and E). Rectal opening is indicated with an arrow. § indicates 0%. Expression of mCherry together with RHO- 1* in the rectal epithelium using the 1.3 Kb *egl-5* promoter fragment (J and K) or infection of animals expressing mCherry from the same promoter (H and I) results in changes in the morphology of the epithelial cells when compared to wild-type controls (F and G). Rectal opening is indicated with an arrow. Rectal epithelial cell boundaries are indicated with a dotted line. P values between 0.05 and 0.001 (*), and P values of 0.001 or less (**).

The simplest explanation for our results is that UNC-73(Trio) activation of RHO-1 is required for immune and behavioral responses to infection. To confirm the requirement for Rho signaling in the rectal epithelial cells we inhibited endogenous RHO-1 in a subset of rectal epithelial cells (K, F and U) by expressing the Rho inhibitor, C3 Transferase, from the *bus-1* promoter and found that this was sufficient to decrease the percentage of Dar animals (Figure 4A). Conversely expression of activated RHO-1(G14V) (RHO-1*) in adults using a heat shock-inducible transgene caused a strong Dar phenotype (Figure 4B, C and D) that was not observed when RHO-1* was expressed from a neuronal promoter (Figure 4B), demonstrating a role for RHO-1 in adult *C. elegans* outside of the nervous system.

Tail swelling was observed when RHO-1* was expressed from a 1.3 Kb *egl-5* promoter fragment that is expressed in the B, K, F, U, and P12.pa rectal epithelial cells and in three posterior body wall muscles [43] (Figure 4B and E) but not when RHO-1* was expressed in the body wall muscles and the B cell using a 469 bp fragment of the same promoter [43] (Figure 4B). Thus, RHO-1 signaling in the adult rectal epithelial cells is sufficient to phenocopy the *C. elegans* response to infection.

How does RHO-1 signaling in the rectal epithelial cells cause the Dar phenotype? One well established role for Rho signaling is the regulation of cell shape [50]. Co-expression of mCherry together with RHO-1* using the same 1.3 Kb *egl-5* promoter fragment allowed us to visualize cell shape changes in rectal epithelial cells. Activation of RHO-1* caused changes in cell morphology; cells appeared larger and were no longer organised around the rectal opening instead spreading towards the dorsal side of the animal (Figure 4 F, G, J and K). These changes were also observed in the rectal epithelial cells of wild-type animals infected with *Microbacterium nematophilum* (Figure 4F–I) [51]. Thus, RHO-1* acts cell-autonomously to alter rectal epithelial cell morphology in a manner similar to the innate immune response to pathogens.

Although inhibition of RHO-1 in a subset of the rectal epithelial cells (the K, F and U cells) using the *bus-1* promoter reduced the Dar response, expression of RHO-1* in these same cells did not trigger the Dar response (Figure 4B) suggesting that coordinated activation of RHO-1 in multiple rectal epithelial cells is required for the Dar response.

### Cholinergic Gaq signaling is required for aversion to pathogenic *M. nematophilum*

**Figure 5:**
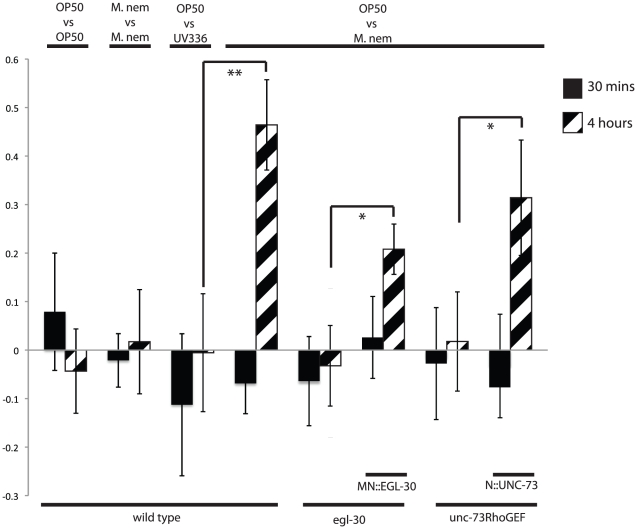
Gαq-Rho GEF Trio-Rho Signaling is required for aversion to pathogenic *M. nematophilum.* Animals were placed equidistant from a two lawns of bacteria (A vs B) and the number of animals on lawns A and B were counted at 30 minutes (solid bars) and at 4 hours (hatched bars). The preference ratio shown is given by the formula [animals at A- animals at B/animals (A+B)]. Wildtype animals have no preference between *E. coli* (OP50) and pathogenic *M. nematophilum* at 30 minutes, but at 4 hours they have a strong preference for OP50 E. coli. This preference is abolished if the strain of *M. nematophilum* is avirulent (UV336) or if animals have a mutation in *unc-73* or *egl-30*. Expression of EGL-30 in the motorneurons (MN::EGL-30) or of UNC-73 in all neurons (N::UNC-73) rescued the preference for OP50 in *egl-30* and *unc-73* mutants respectively. P values between 0.05 and 0.001 (*), and P values of 0.001 or less (**).

What is the physiological effect of increases in locomotion in response to infection by *M. nematophilum*? Previous results have shown that if given a choice between lawns of *E. coli* and *M. nematophilum* then after 4 hours *C. elegans* have left lawns of *M. nematophilum*, and this is termed the aversion behavior. We have repeated these experiments and show that animals do avoid *M. nematophilum* but do not avoid the avirulent *M. nematophilum* strain UV336 suggesting that aversion requires infection of *C. elegans* (Figure 5). We also noticed that initially, after 30 minutes, animals show no aversion to *M. nematophilum* suggesting that aversion differs to that of repellents such as quinine, to which *C.elegans* responds to in seconds [52]. The *M. nematophilum* aversion behavior was lost in animals with mutations in EGL-30 (Gαq) (*egl-30(ad805)*) or UNC-73 (Trio) (*unc-73(ce362)*). Expression of EGL-30 in cholinergic motorneurons (MN::EGL-30) partially rescued the aversion behavior of the *egl-30(ad805)* mutants suggesting that at least some of the aversion response occurred independent of EGL-30 (Gαq) signaling in the sensory neurons (Figure 5). Expression of UNC-73 from pan-neuronal promoter (N::UNC-73) rescued the aversion behavior of the *unc-73(ce362)* mutants demonstrating that neuronal RHO-1 signaling is required for aversion behavior.

### Gαq/Rho and Ras converge on Raf/MEK/ERK signaling during the immune response

It was previously shown that the Raf/MEK/ERK MAPK pathway is necessary and sufficient for the Dar response: hyper activation of the pathway by the over expression of constitutively active forms of LIN-45 (Raf), MEK-2 (MEK), or MPK-1 (ERK) results in tail swelling in the absence of infection (as we have shown for constitutively active EGL-30* and RHO-1*), while mutations in *lin-45*, *mek-2* or *mpk-1* result in a defective Dar response [42]. Blocking MAPK signaling using the MEK inhibitor U0126, RNAi for *mpk-1*, or mutations in *lin-45(sy96)*, *mek-2(n1989)*, or *mpk-1(ku1)* significantly decreased the Dar response induced by RHO-1*. (Table 2 and Figure S2). In addition, we observed that loss of RHO-1 signaling, using an *unc-73(ce362)* mutant, was unable to suppress Dar induced by over expression of constitutively activated LIN-45, MEK-2, or MPK-1 (Table 2), demonstrating that Rho signaling acts upstream of Raf and its downstream effectors the MAPKs to trigger the Dar response.

**Table 2:** Analysis of genetic interactions between RHO-1 and the Ras/MAP Kinase pathway.

Effect of Ras/MAP Kinase pathway inhibition on RHO-1*-induced *dar* phenotype		
Genotype	% animals with *dar* phenotype ± s.e.m	n value
hs::RHO-1*	90.2±4.5	13
hs::RHO-1*+DMSO	81.±5.5	3
hs::RHO-1*+50 µM U0126	0±0^a^	3
hs::RHO-1*+control RNAi	90.4±1.6	19
hs::RHO-1*+*mpk-1* RNAi	25.8±15.6^b^	3
hs::RHO-1*; *lin-45(sy96)*	26.4±6.0^c^	7
hs::RHO-1*; *mek-2(n1989)*	2.5±2.5^c^	3
hs::RHO-1*; *mpk-1(ku1);unc-32(e189)*	0±0^c^	3
*egl-5p*::RHO-1*	83.8±1.8	4
*egl-5p*::RHO-1*+DMSO	78.3±4.7	9
*egl-5p*::RHO-1*+50 µM U0126	23.8±3.2^d^	9
*egl-5p*::RHO-1*+control RNAi	78.0±3.3	9
*egl-5p*::RHO-1*+*mpk-1* RNAi	31.9±3.4^e^	9
*egl-5p*::RHO-1*; *let-60(n2021)*	31.9±4.5^f^	4
*egl-5p*::RHO-1*; *lin-45(sy96)*	24.4±4.4^f^	5
*egl-5p*::RHO-1*; *mek-2(n1989)*	0±0^f^	4
*egl-5p*::RHO-1*; *mpk-1(ku1);unc-32(e189)*	0±0^f^	4
**Effect of ** ***unc-73(ce362)*** on Ras/MAP Kinase*-induced ***dar*** ** phenotype**		
EF1a::DMEK;hs::MPK-1	87.3±1.2	5
EF1a::DMEK;hs::MPK-1;*unc-73(ce362)*	79.6±2.8	5
hs::MEK-2*	85.9±5.8	5
hs::MEK-2*;*unc-73(ce362)*	87.8±4.2	5
hs::LIN-45*	53.9±4.6	5
hs::LIN-45*;*unc-73(ce362)*	52.5±7.6	5
hs::LET-60*	71.6±2.7	5
hs::LET-60*;*unc-73(ce362)*	34.5±4.4^g^	5
hs::LET-60*+DMSO	66.4±2.2	3
hs::LET-60*+50 µM U0126	24.4±4.4^h^	3

The number of *dar* animals was scored as a percentage of the total. Heatshock, drug treatment or RNAi were as described in the Methods. All animals containing heat shock transgenes were heat shocked as described in the Methods, no *dar* phenotype was observed in unheatshocked controls. Values are means +/− the standard error. No *dar* animals were observed in *mpk-1(ku1);unc-32(e189), mek-2(n1989), lin-45(sy96), let-60(n2021)* or *unc-73(ce362)* single mutants.

a = p<0.001 relative to hs::RHO-1*+DMSO.

b = p<0.001 relative to hs::RHO-1*+control RNAi.

c = p<0.001 relative to hs::RHO-1*.

d = p<0.001 relative to *egl-5p*::RHO-1*+DMSO.

e = p<0.001 relative to hs::RHO-1*+control RNAi.

f = p<0.001 relative to *egl-5p*::RHO-1*.

g = p<0.001 relative to hs::LET-60*.

h = p<0.001 relative to hs::LET-60*+DMSO.

**Figure 6:**
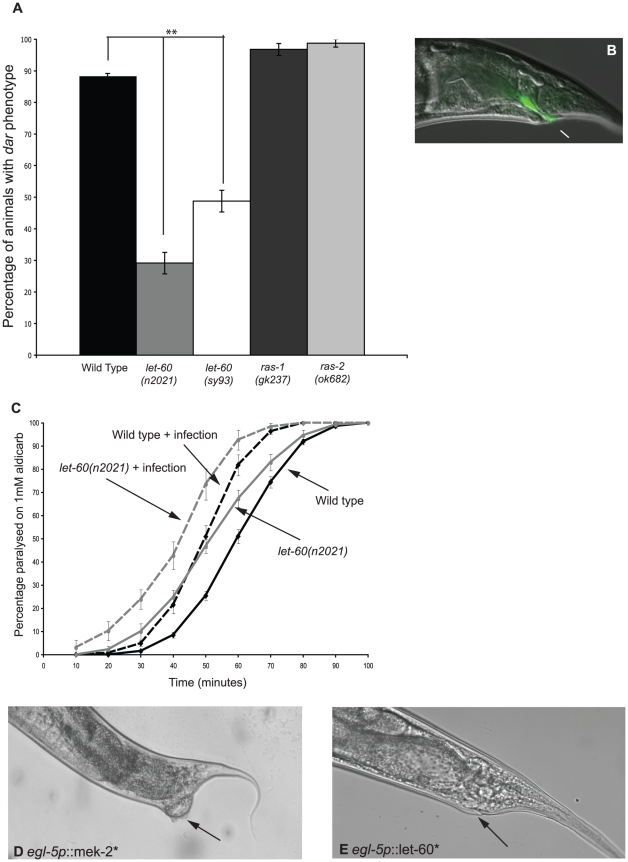
LET-60 (Ras) activation is sufficient to cause tail swelling and is required for the Dar response to infection. Two different *let-60* (Ras) reduction-of-function mutations, *n2021* and *sy93,* significantly decreased the Dar response upon infection with *M. nematophilum* (A). This decrease was not observed using *ras-1(gk237)* and *ras-2(ok628)* mutants that showed a wild-type response to infection (A). Although decreased tail swelling was observed in *let-60(n2021)* animals infected with *M. nematophilum* bacteria, labeled using the nucleic acid stain SYTO13, still attached to the anal opening (B). *let-60(n2021)* animals were slightly hypersensitive to aldicarb when grown on *E. coli* OP50 and ACh release was increased in these animals following infection with *M. nematophilum* (C). Cell specific expression of constitutively active MEK- 2(S223E, S227D) (*egl-5p*::MEK-2*) (D) or constitutively active LET-60(G12V) (*egl- 5p*::LET-60*) (E) in the rectal epithelial cells using a 1.3 Kb *egl-5* promoter fragment resulted in tail swelling that phenocopied the Dar phenotype observed following infection and RHO-1* activation. Arrows in D and E indicate the rectal opening. P values between 0.05 and 0.001 (*), and P values of 0.001 or less (**).

The small GTPase Ras activates the ERK MAPK pathway and, in mammalian cells, RhoA cooperates with Ras during cell transformation [53]. Therefore, we tested whether the *C. elegans* Ras genes (*let-60, ras-1,* and *ras-2*) and RHO-1 cooperate during the Dar response. RHO-1*-induced Dar significantly decreased in animals with a reduction-of-function mutation in *let-60(n2021),* indicating that RHO-1 acts either upstream, or in parallel to, LET-60 (RAS) during the Dar response (Table 2). Previous studies have reported a wild-type response to infection in *let-60(n2021)* mutants [42], however, we observed that the Ras mutants *let-60(n2021)* and *let-60(sy93)* had a reduced Dar response when exposed to *M. nematophilum* (Figure 6A). *let-60(n2021)* decreased *M. nematophilum-*induced tail swelling, however, bacteria (labelled with SYTO13) were still observed adhering to the rectum (Figure 6B) demonstrating that mutations in LET-60 (RAS) do not block infection but do block the Dar response. Mutations in the other Ras genes, *ras-1(gk237)* or *ras-2(ok628)*, had no effect, suggesting that LET-60 (RAS) is the only RAS gene required during the Dar response triggered by infection or RHO-1* activation (Figure 6A).

Interestingly although Ras (LET-60) signaling is required for the immune response to infection it is not required for the behavioral response. Although *let-60(n2021)* animals were slightly hypersensitive to aldicarb when grown on *E. coli* OP50, infection by *M. nematophilum* increased ACh release in *let-60(n2021)* mutants in a manner similar to that observed in wild type controls (Figure 6C).

In *C. elegans* the function of LET-60 (RAS) has been best characterized during vulval formation, where a gain-of-function mutation in the chromosomal *let-60(n1046)* results in a multi-vulval phenotype [54]. Over expression of constitutively active LET-60(G12V) (LET-60*), either in adult animals (using the heat shock-inducible promoter) or in the rectal epithelium, did cause the Dar response (Figure 6D), however, the chromosomal gain-of-function *let-60(n1046)* mutation was not sufficient to trigger the Dar response (Table 1). Expression of constitutively active LET-60(RAS) in rectal epithelial cells did not cause as pronounced a Dar response as observed with constitutively active MEK-2 from the same promoter (Compare Figure 6D and E) and this could reflect differences in expression of the genes, the strength of the activating mutation or differences in the numbers of downstream LET-60(RAS) pathways activated.

Using the MEK inhibitor U0126 we were able to suppress the Dar response induced by either RHO-1* or LET-60* (Table 2 and Figure S2) indicating that both of these pathways act upstream of RAF/MEK/ERK to mediate the Dar response and suggesting that at least one signal required for Raf activation in the rectal epithelia is RHO-1 dependent. Consistent with this the LET-60*-induced Dar response was significantly decreased in an *unc-73(ce362)* mutant (Table 2). The Dar response of the LET-60*; *unc-73(ce362)* animals was further reduced by addition of U0126 but this was not a significant change (p = 0.18). Our results could suggest that RHO-1 acts downstream of LET-60 (RAS), but a mutation in *x* blocked the Dar response caused by RHO-1* (Table 2) suggesting the RHO-1 and LET-60(RAS) pathways act in parallel. Both RHO-1 and LET-60 (RAS) act upstream of Raf/MEK/ERK so our data suggests that these parallel pathways converge on Raf (*lin-45*) to regulate ERK/MAPK signaling and trigger the Dar response during the innate immune response to pathogenic bacteria.

### Activation of multiple signaling pathways is required to trigger the immune response

Both the Gαq-RhoGEF Trio-RHO-1 and Ras signaling pathways act upstream of LIN-45 (Raf) to mediate the immune response to infection, however, chromosomal gain-of-function mutations in EGL-30 (Gαq) or LET-60 (Ras) were not Dar suggesting that levels of signalling from these mutations was not individually sufficient to trigger the immune response. To investigate whether simultaneous activation of these pathways was able to cause the Dar phenotype we over expressed constitutively active EGL-30(Q205L) in the chromosomal gain-of-function *let-60(n1046)* mutant and observed an increase in the number of Dar animals when compared to expression of this transgene in wild type animals (Table 1). In addition we observed a number of animals with the Dar phenotype when we combined the chromosomal gain-of-function mutations in both *egl-30(js126)* and *let-60(n1046)* (Table 1). These two observations suggest that these pathways act in parallel to cause the Dar phenotype perhaps acting as a coincidence detector between two infection signals. However, in both of these experiments the increase in Dar animals was small suggesting that in wildtype animals additional factors are required to mediate a robust Dar response to *M. nematophilum* infection.

## Discussion

### An EGL-30 (Gαq) signaling pathway is required for both behavioral and innate immune response to infection

*C. elegans* display both behavioral and innate immune responses upon exposure to pathogenic *M. nematophilum* [9], [23]. Avirulent strains of *M. nematophilum* fail to induce immune responses that include the Dar response [9] and expression of putative anti-microbial peptide genes [36], [39] and here we show that the avirulent UV336 strain of *M. nematophilum* is also unable to trigger behavioral responses. The failure of the avirulent UV336 strain to increase locomotion or increase ACh release compared to the *E. coli* control makes it unlikely that behavioural responses are due to different nutritional values of *M. nematophilum* versus *E. coli,* i.e. animals growing on *M. nematophilum* receive less nutrition relative to animals growing on *E. coli*. The simplest explanation for these results is that *C. elegans* is capable of recognizing that it has become infected and coordinates behavioural and immune responses in response.

What are the signals produced by infection? Both the behavioral and innate immune response to infection require the conserved EGL-30 (Gαq)/UNC-73 (Trio RhoGEF)/RHO-1 (RhoA) signaling pathway (henceforth referred to as the EGL-30 (Gαq) pathway). Defects in the EGL-30 (Gαq) pathway do not prevent infection by *M. nematophilum,* instead activation of the EGL-30 (Gαq) pathway is required in neurons and the rectal epithelial cells to trigger behavioral and Dar responses to infection respectively. Here we have only addressed locomotion, ACh release, aversion and the Dar response but infection can also triggers other changes, for example expression of anti-microbial peptides [36], and the EGL-30 (Gαq) pathway could play a role in coordinating a wider range of responses to pathogen than studied here. Indeed, EGL-30 (Gαq) is required in the intestine for protection against *P. aeruginosa*, although it is unknown if RHO-1 signaling is also required [55]. Thus, our results demonstrate that in response to infection signals that activate Gq coupled GPCRs are at some point required.

### EGL-30 (Gαq), UNC-73 (Trio RhoGEF) and RHO-1 (RhoA) act in different cell types to mediate behavioral and Dar responses to *M. nematophilum*

In which cells are the Gq coupled GPCRs that trigger behavioral and immune responses to infection located? Cell specific rescue experiments show that cholinergic EGL-30 (Gαq) signalling is required for behavioural responses to infection whereas rectal epithelial EGL-30 (Gαq) signalling is required for the Dar response to infection. We have previously demonstrated a role for EGL-30 (Gαq) signalling in cholinergic neurons [29], [31] and here we show that one mechanism by which the cholinergic EGL-30 (Gαq) pathway is activated is in response to infection. In the case of the immune response this is the first demonstration of a role for EGL-30 (Gαq) signalling in rectal epithelial cells for the Dar response. The rectal epithelial cells consist of five cells (B, F, Y, U, and K′), expression of activated RHO-1 in all five cells caused a Dar response in the absence of infection, whereas expression only in B failed to trigger the response. In contrast, inactivation of RHO-1 in just the B cell prevented a Dar response upon infection. Thus, coordinated EGL-30 (Gαq) signalling in multiple, if not all, rectal epithelial cells is required for the Dar response. Our results demonstrate separate sites of action for the EGL-30 (Gαq) signaling pathway in behavioral and immune responses to infection and argue against a model in which EGL-30 (Gαq) signaling acts in a single cell to produce further secreted signals that go on to trigger behavioral and immune responses to infection. Our data also argues against a model whereby the Dar response triggers behavioral changes and *vice versa*. We conclude that Gq coupled GPCRs present on the cholinergic motorneurons and on multiple rectal epithelial cells are required for the behavioral and immune responses of *C. elegans* respectively in response to infection by *M. nematophilum.*

### EGL-30 (Gαq) signaling in the motorneurons mediates the aversive response to *M. nematophilum*

What is the physiological relevance of the behavioral response to infection by *M. nematophilum?* We show that *C.elegans*, when infected by *M. nematophilum,* move faster and we show that this results in the animals leaving a lawn of *M. nematophilum.* Such a response is likely to lessen exposure to *M. nematophilum* and subsequent eggs laid will not hatch in the presence of pathogen. We also believe the behavioural changes we observe in response to infection explain the aversion of *C.elegans* to lawns of *M. nematophilum.* Yook et al. first demonstrated that given a choice between lawns of *E.coli* and *M. nematophilum* animals preferentially localized to the *E. coli* lawn after 4 hours [23] and this is termed the aversive response. Our results are consistent with the behavioral responses we report here as playing an important part in the aversive response. Firstly, neither the behavioral or aversive responses are triggered by the avirulent *M. nematophilum* strain UV336. Secondly, both responses fail to occur in EGL-30 (Gαq) and UNC-73 (TrioRhoGEF) mutant animals. And thirdly, these responses are rescued by cholinergic motorneuron expression of EGL-30 and neuronal expression of UNC-73. Evidence from aversive responses to other pathogens suggests that aversion can be a learnt response requiring both chemosensory neurons and interneurons [22], [56]. We observe that *C. elegans* do not avoid lawns of *M. nematophilum* after 30 minutes but do so after 4 hours and this is consistent with, but does not prove, a learnt behavior. Our rescue experiments suggest that if chemosensory and interneurons are required for aversion to *M. nematophilum* then the pathways acting within those neurons can signal in the absence of EGL-30 (Gαq) signaling. However, the partial rescue of the aversion response by cholinergic motorneuron expression EGL-30 (Gαq) could indicate that the full aversion response does require additional EGL-30 (Gαq) signaling in other cells, for example the sensory neurons. Mutations in two components of a cyclic nucleotide gated channel, *tax-2* and *tax-4*, also prevent aversion of *C.elegans* to lawns of *M. nematophilum* [23]. *tax-2* and *tax-4* genes are required in sensory neurons to mediate aversive responses to *S. marcescens* [19] possibly suggesting that chemosensory neurons are also required for aversion to *M. nematophilum*. However, *tax-2* and *tax-4* mutants also fail to produce the Dar response to *M. nematophilum* infection and they have been reported to have cuticle defects [23] thus, currently we cannot determine if sensory neurons are required for the aversive or Dar response to *M. nematophilum*.

### RHO-1 or LET-60 (Ras) converge on LIN-45 (Raf) to trigger the Dar innate immune response

Previously it has been shown that a conserved LIN-45 (Raf)/MEK-2 (MEK)/MPK-1 (ERK) MAPK pathway is required for the Dar response [42]. Raf is activated by Ras GTPases in other systems and here we have shown that LET-60 (Ras) mutations blocked the Dar response to pathogen whereas transgenic overexpression of activated LET-60 (RAS) in the rectal epithelial cells triggered the Dar response. As with the EGL-30 (Gαq) pathway, signalling by LET-60 (Ras), LIN-45 (Raf), MEK-2 (MEK) and MPK-1 (ERK) (hereafter referred to as the LET-60 (Ras) pathway) is required cell autonomously within the rectal epithelial cells for the Dar response. Two results suggest that within the rectal epithelial cells the EGL-30 (Gαq) pathway and LET-60 (Ras) converge on LIN-45 (Raf) to trigger the Dar response – Firstly, reductions in signaling of the MAPK pathway using either mutations in LIN-45 (Raf), MEK-2 (MEK), MPK-1 (ERK), or chemical inhibition of MEK-2 (MEK) using U0126 blocked the Dar response triggered by transgenic expression of activated RHO-1 or LET-60 (RAS). In contrast, mutations in RHO-1 signaling (UNC-73 (Trio)) or LET-60 (Ras) did not block the Dar response triggered by transgenes expressing gain-of-function mutations in LIN-45 (Raf), MEK-2 (MEK), and MPK-1 (ERK). These results suggest that both RHO-1 and LET-60 (RAS) act upstream of LIN-45 (Raf).

Secondly, a mutation in UNC-73 (RhoGEF) blocked the Dar response triggered by transgenic expression of activated LET-60 (RAS), and a mutation in LET-60 (RAS) blocked the Dar response triggered by transgenic expression of activated RHO-1. Thus, for the Dar response, defects in RHO-1 or LET-60 (Ras) signaling co-suppressed each other suggesting that these two pathways act in parallel.

**Figure 7:**
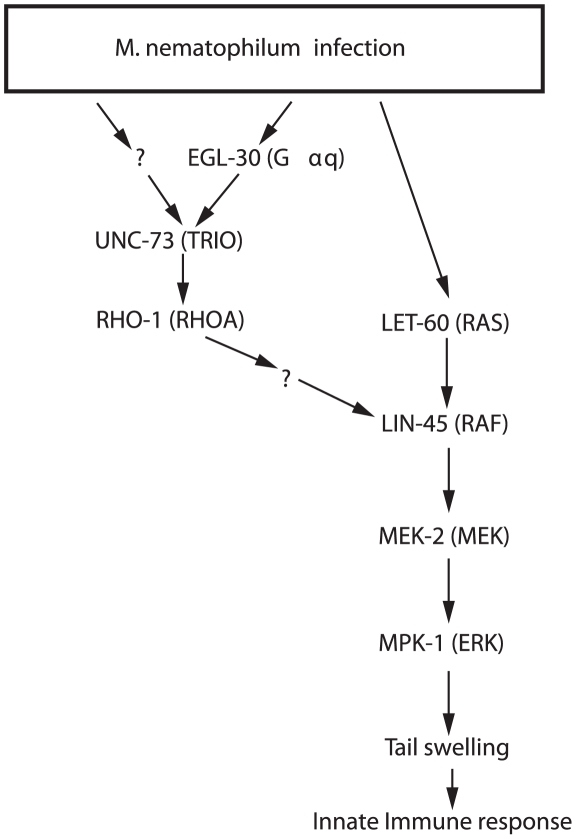
Gαq-Rho GEF Trio-Rho Signaling and Ras converge on Raf to regulate morphology during the immune response to infection. The simplest explanation of our results is that following pathogen infection RHO-1 is activated in the rectal epithelial cells by multiple upstream regulators including EGL-30 (Gαq) and UNC-73 (Trio). Together with Ras, Rho signaling converges on Raf to activate the MAPK pathway. Activation of these pathways, together with at least one other, in the rectal epithelial cells leads to the changes in morphology that occur as part of the immune response.

The simplest model that explains our data is that in rectal epithelial cells the RHO-1 and LET-60 (Ras) signaling pathways converge on LIN-45 (Raf) (Figure 7). The requirement for convergent RhoA and Ras signaling for Raf activation has also been observed in mammalian cells, where dominant negative forms of RhoA blocked the ability of Ras to activate Raf, indicating that Rho signaling is required for Raf activation, although the mechanism is unknown [57]. Alternative interactions between Rho and Ras also exist. During *C. elegans* vulval formation RHO-1 appears to act upstream of LET-60 (Ras) [54] suggesting that the Rho and Ras signaling pathways can either act in parallel or in series depending on the cell type. Interactions between Rho and Ras pathways appear to be essential during cellular transformation [53] and co-activation of RhoA and Ras signaling can lead to different responses from those signaled by either pathway alone [58]. It will be important to identify the cell specific factors that control the interactions between the Rho and Ras signaling pathways. Infection of *C. elegans* with *M. nematophilum* provides a starting point for genetic screens to identify the molecular mechanisms by which RhoA and Ras act together to activate Raf. The factors that allow this co-operation are likely to be critical in *C. elegans* and mammals for signaling involved in innate immunity and oncogenesis. These results also demonstrate that in wildtype animals the Dar response requires two signals: one that activates GPCRs coupled to the EGL-30 (Gαq) pathway and a second that activates receptors that activate the LET-60 (RAS) pathway. Where are these signals likely to be produced? One candidate is the hypodermal cells that are the focus of the *M. nematophilum* infection. Hypodermal signaling is required to induce expression of anti-microbial peptides in response to infection by *D. coniospora* [59], [60], which, like *M. nematophilum,* infects the hypodermis. This hypodermal signaling requires p38 MAPK signaling [59] and it will be interesting to test if the response to *M. nematophilum* also requires activation of the p38 MAPK pathway in hypodermal cells. Identifying the ligands that activate the receptors coupled to EGL-30 (Gαq) and LET-60 (Ras) will provide important clues to how *C.elegans* recognises it has been infected.

### Innate immune responses are harder to trigger than behavioral responses

Transgenic activation of genes in the EGL-30 (Gαq) and LET-60 (Ras) pathways led to the Dar response even in the absence of pathogen, however, gain-of-function mutations in the endogenous chromosomal genes did not cause the Dar response. For example the *egl-30(js126gf)* gain-of-function mutation increased locomotion and ACh release [44] but did not trigger the Dar response and the *let-60(n1046gf)* gain-of-function mutation results in multiple vulva formation [61] but did not trigger the Dar response. A small percentage of animals with both the *egl-30(js126gf)* and *let-60(n1046gf)* mutations did show a Dar response in the absence of pathogen but at a much lower rate than observed following transgenic expression of gain-of-function mutants of EGL-30 (Gαq) and LET-60 (RAS). The *n1046gf* mutation causes a Gly to Glu change in LET-60 (RAS) at a position 13 [62], an amino acid change known to cause oncogenic activation in mammalian RAS [63], [64]. The *js126gf* mutation causes a Val to Met change in EGL-30 (Gαq) at position 180 [65] and this mutation is predicted to interfere with the GAP activity of EGL-30 (Gαq). Both the *n1046gf* and *js126gf* mutations are semi-dominant [61], [62], [65] but the amino acid changes involved differ from the mutations used to cause constitutive activation in our transgenes (EGL-30(Q205L) and LET-60(G12V)) and it is unclear to what level these different mutations activate EGL-30 (Gαq) and LET-60 (Ras). It appears likely that our transgenes cause higher levels of EGL-30 (Gαq) and LET-60 (Ras) signalling, either the mutations involved in the transgenes cause stronger activation, the increased level of expression from the transgenes results in stronger signaling, or a combination of these two possibilities. Perhaps strongly activating mutations in the chromosomal EGL-30 (Gαq) and LET-60 (RAS) genes cause lethality, whereas restricted expression of strongly activating EGL-30 (Gαq) and LET-60 (RAS) mutations from a transgene can be tolerated.

Unlike the Dar response, both transgenic and chromosomal gain-of-function mutations in EGL-30 (Gαq) are sufficient to trigger changes in ACh release and locomotion, and these neuronal changes do not require inputs from the LET-60 (RAS) pathway. This suggests that the Dar response requires a higher level of EGL-30 (Gαq) signaling than the behavioral response to infection. In addition the Dar response requires coincident EGL-30 (Gαq) and LET-60 (RAS) signaling and this is not apparently required for the behavioral response to infection. Perhaps the consequences of inappropriate activation of the innate immune response are more severe than inappropriate activation of the behavioral response and animals may set a higher threshold for the Dar response than changes in behavior.

### *C. elegans* as a model to study coordinated neuronal and immunological response to infection

This is the first demonstration for a role for RHO-1 in *C. elegans* innate immunity, however, its mammalian ortholog RhoA is a key regulator of mammalian immune responses acting to regulate Toll receptor signaling, leukocyte migration, and phagocytosis of pathogens [66] suggesting further parallels between mammalian and *C. elegans* innate immunity. Although less well studied than the immune response behavioral changes following infection play an important role in defending many species, including humans, from pathogen attack [18]. Coordination of these responses makes sense as it allows animals to mount an immune response to the immediate threat whilst simultaneously taking action to remove the pathogen, however, the complicated nature of the mammalian brain and immune system has made it difficult to identify the molecular mechanisms that mediate these interactions. With its simple, well described, nervous system and a rapidly growing understanding of its immune system, *C. elegans* provides a model to understand the role RhoA and Gαq signaling play in coordinating behavioral and immune responses to infection [67].

## Materials and Methods

### Strains

*C. elegans* strains used in this study are detailed in Supplemental material. All strains were cultivated at 20°C on nematode-growth media (NGM) plates seeded with *E. coli* OP50 unless otherwise stated and maintained as described previously [68].

### Transgenes and germline transformation

Plasmids (listed as pRJM or SJN) were constructed using standard techniques, and verified by sequencing. Transgenic strains (listed as *nzEx* or *impEx*) were isolated by microinjection of 100 ng/µl of plasmid unless otherwise described below together with *ttx-3::gfp* (a gift of O. Hobert, Columbia University NY), *unc-122::gfp* (a gift of P. Sengupta Brandeis University MA), *rol-6* dominant marker, or *acr-2::mcherry* (SJN445) at 50 ng/µl as a marker. Some cDNAs were obtained from Yuji Kohara at the Center for Genetic Resource Information, National Institute of Genetics, Research Organization of Information and Systems, Mishima, Japan. Unless otherwise stated all injections were performed into N2 animals. Plasmids and transgenic strains are described in Supplemental Methods.

### *M. nematophilum* infection and staining

Infection with *M. nematophilum* was performed as described previously [42] with the following modifications. NGM plates were seeded with 10% *M. nematophilum* diluted in OP50 *E. coli*. Adult animals were transferred to infection plates and were maintained at 20°C or 25°C. F1 progeny were scored for the presence or absence of the Dar phenotype once they reached L4 or adult stages. In the case of hs::UNC-73;*unc-73(ce362)* animals synchronized populations of L1 animals were obtained by bleaching and these L1′s were transferred to infection plates. This generation was assayed for the presence of the Dar phenotype. SYTO13 staining was performed as described previously [42].

### Analysis of locomotion and sensitivity to drug treatment

Adult animals were infected with 10% *M. nematophilum* diluted in OP50 *E. coli* and F1 progeny were assayed as one-day-old adults. Locomotion assays were performed as described previously [69]. Sensitivity to 1 mM aldicarb (Greyhound Chromatography) was determined by analysing the onset of paralysis as described previously [40]. For each experiment, at least 20 animals were tested and each experiment was repeated at least four times. Error bars indicate the s.e.m.

### Aversion assay

Assays were performed essentially as described by Yook et al. (2007) with the following changes. Assays were performed on 60 mm plates with 40 µl of an overnight culture of bacteria grown in LB placed on opposite sides of the plate. Animals were washed in M9 and allowed to settle before aspiration, centrifugation of the animals was found to alter their behavior and was not used. A suspension of animals in a drop of M9 was placed equidistant from each bacterial lawn, numbers of animals varied from 25 to 100. The chemotaxis index = (number of animals on lawn A- number of animals on lawn B)/number of animals on lawn A+B. In all experiments lawn A was OP50 except where both lawns contained *M. nematophilum.*

### Induction of heat shock-inducible transgenes

Expression from the heat shock promoter was achieved using two rounds of heat shock for 60 min separated by 30 min at 20°C. Heat shock was performed on one-day-old adults or L4′s except for in hs::UNC-73E;*unc-73(ce362)* animals where heat shock was performed at 0, 24 and 48 hours after transfer to *M. nematophilum* plates when animals were at approx L1, L2/3 and L3/4 stage respectively. For transgenic animals containing hs::RHO-1* or hs::UNC-73E transgenes a heat shock temperature of 33°C was used. For all other transgenes heat shock was performed at 37°C. Animals were allowed to recover overnight at 20°C before scoring for the Dar phenotype.

### MAPK inhibition using U0126

One-day-old adults were transferred to NGM plates seeded with OP50 containing 50 µM U0126 (Sigma) or DMSO (as a control). Plates were incubated at 20°C for 2 hours and animals were heat shocked as described above. Animals were allowed to recover overnight at 20°C before scoring for the Dar phenotype.

### Microscopy

Animals were imaged by mounting on 2% agarose pads. DIC images were obtained using a Zeiss Axioplan microscope with ×40 objective. Digital images were captured using Openlab software (Improvision) and processed using ImageJ (NIH). For fluorescence microscopy animals were viewed on a Leica TCS SP5 microscope with a Leica ×63 objective. Images were obtained using Leica Application Suite Microscope software. Digital images were processed to give maximum intensity projections or 3D projections of a Z-series using ImageJ (NIH).

### Statistical analysis

In all cases statistical analysis was performed using an unpaired two-tailed t-test. P values between 0.05 and 0.001 (significant) are indicated on figures using one asterisk, and P values of 0.001 or less (highly significant) are indicated with two asterisks.

### Ethics statement

No vertebrate animals were used for these studies and no ethical approval was required.

## Supporting Information

Figure S1 **EGL-30 (Gαq) signaling in the rectal epithelium fails to rescue severe constipation in infected** ***egl-30(ad805)*** **animals.** A. Uninfected *egl-30(ad805)* adult animals. An asterisk indicates the intestine. B. *egl-30(ad805)* animals infected with *M. nematophilum* are *bus* and severely constipated. C. Expression of EGL-30 (Gαq) in the rectal epithelial cells using a 1.3 Kb *egl-5* promoter fragment rescues the Dar phenotype following infection however these animals remain severely constipated. Extent of intestinal distention is indicated by double-headed arrows.
(TIF)

Figure S2 Inhibition of the MAPK pathway suppresses the RHO-1* induced Dar. Adult wild-type animals and animals expressing hs::RHO-1* were pre-treated with 50 µM of the MEK inhibitor U0126 (or DMSO as a control) for 2 hours at 20°C and then heat shocked as described in Material and Methods. After overnight recovery the percentage of animals showing the Dar phenotype was scored. No Dar response was observed in wild-type animals treated with either DMSO or U0126 (A and B). Animals expressing activated RHO-1* were Dar (C) and this was blocked by pre-treatment with U0126 (C and D). Rectal opening is indicated with an arrow.
(TIF)

Protocol S1 **Details of plasmids and strains used.**
(DOC)
