# Supplementary figures and images for: Behavioral and Immune Responses to Infection Require Gαq- RhoA Signaling in C. elegans
Source: PLoS Pathog. 2012 Feb 16;8(2):e1002530. doi: 10.1371/journal.ppat.1002530 (PMC3280986; doi:10.1371/journal.ppat.1002530)

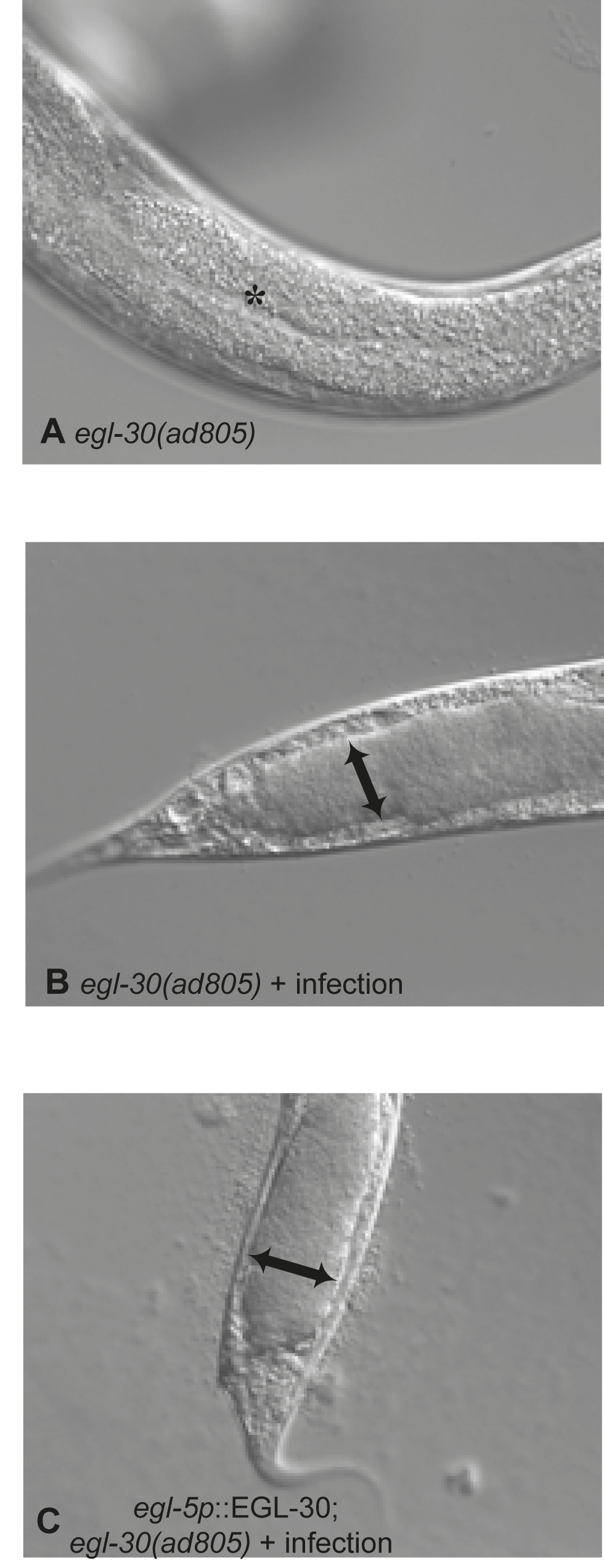

Supplement: Figure S1 — EGL-30 (Gαq) signaling in the rectal epithelium fails to rescue severe constipation in infected egl-30(ad805) animals. A. Uninfected egl-30(ad805) adult animals. An asterisk indicates the intestine. B. egl-30(ad805) animals infected with M. nematophilum are bus and severely constipated. C. Expression of EGL-30 (Gαq) in the rectal epithelial cells using a 1.3 Kb egl-5 promoter fragment rescues the Dar phenotype following infection however these animals remain severely constipated. Extent of intestinal distention is indicated by double-headed arrows. (TIF) [file ppat.1002530.s001.tif]

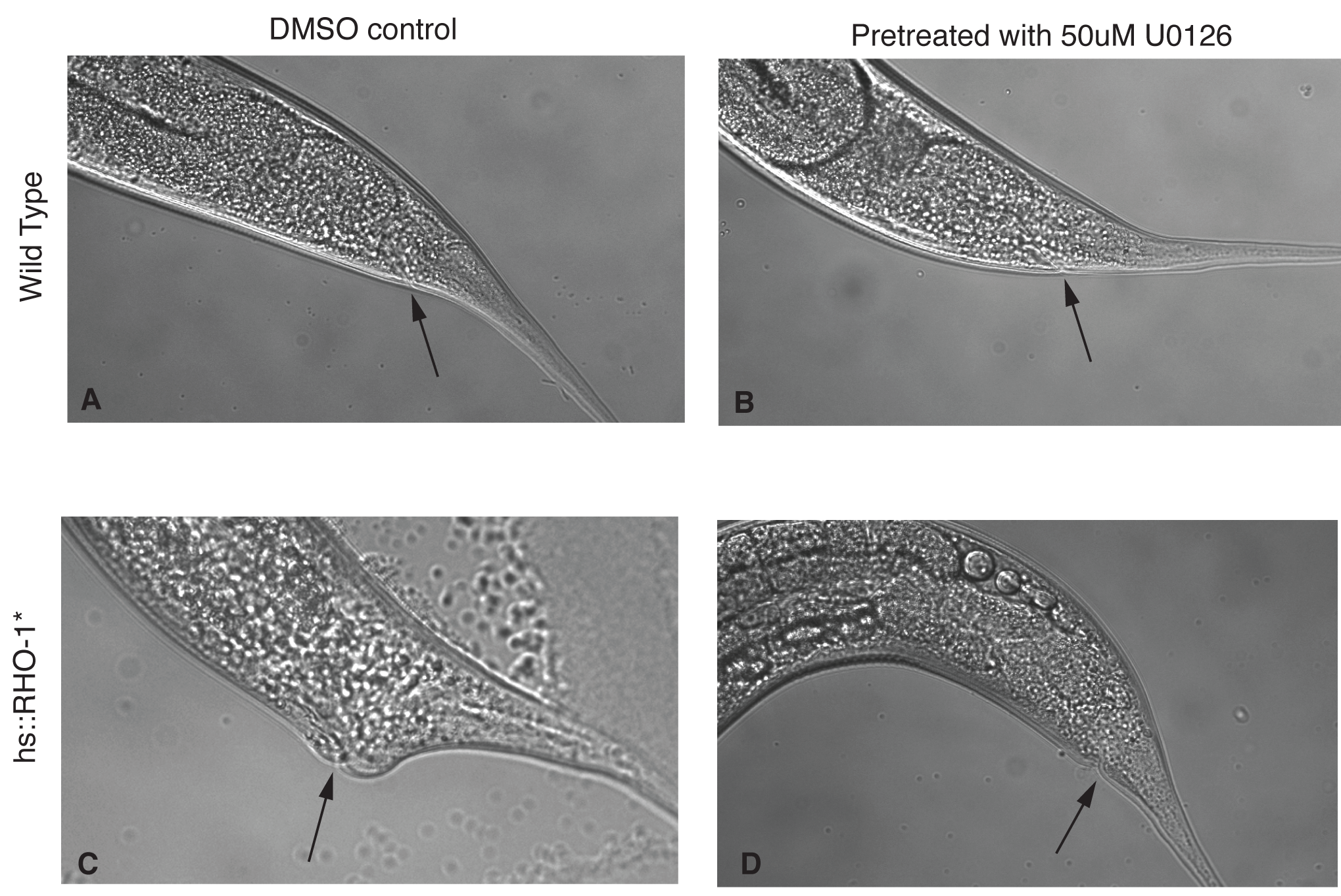

Supplement: Figure S2 — Inhibition of the MAPK pathway suppresses the RHO-1* induced Dar. Adult wild-type animals and animals expressing hs::RHO-1* were pre-treated with 50 µM of the MEK inhibitor U0126 (or DMSO as a control) for 2 hours at 20°C and then heat shocked as described in Material and Methods. After overnight recovery the percentage of animals showing the Dar phenotype was scored. No Dar response was observed in wild-type animals treated with either DMSO or U0126 (A and B). Animals expressing activated RHO-1* were Dar (C) and this was blocked by pre-treatment with U0126 (C and D). Rectal opening is indicated with an arrow. (TIF) [file ppat.1002530.s002.tif]
