# Supplementary material for: Behavioral and Immune Responses to Infection Require Gαq- RhoA Signaling in C. elegans
Source: PLoS Pathog. 2012 Feb 16;8(2):e1002530. doi: 10.1371/journal.ppat.1002530 (PMC3280986; doi:10.1371/journal.ppat.1002530)
Supplement: Protocol S1 — Details of plasmids and strains used. (DOC) [file ppat.1002530.s003.doc]

**SUPPLEMENTAL PROTOCOLS**

**Plasmid and transgene construction**

**EGL-5 promoter Transgenes.** pLG7 (a gift of P. Sternberg, Caltech CA) and pHN5c (a gift of J. Hodgkin, Oxford University UK) contain fragments of the *egl-5* promoter upstream of the *pes-10* minimal promoter. pLG7 contains a 1.3Kb fragment of the *egl-5* promoter that drives nuclear localized GFP expression in B, K, F, U, P12.pa and three body wall muscles in the posterior (Teng et al. 2004). pHN5c contains a 469bp *egl-5* promoter fragment that has been reported to drive expression in the B cell and body wall muscles (Nicholas and Hodgkin 2009). The 1.3Kb *egl-5p* fragment together with the *pes-10* promoter was subcloned upstream of mCherry (pRJM088). This plasmid was injected together with pLG7 at 20ng/μl and *nzIs38* contains an integrated version of pRJM088.

**RHO-1 Transgenes.** The RHO-1 cDNA was isolated and modified to create the constitutively active G14V mutation as described previously (McMullan et al. 2006). The *egl-5* promoter fragments and *pes-10* minimal promoters from pLG7 and pHN5c were inserted upstream of the RHO-1(G14V) cDNA to give pRJM093 and pRJM115 respectively. These plasmids were injected at 5ng/μl. *nzEx483* and *nzEx505* contain extrachromosomal versions of pRJM093 and pRJM115 respectively. The *bus-1* promoter that drives expression in K, F and U cells (Gravato-Nobre and Hodgkin 2008) was taken from pMGN7 (a gift from J. Hodgkin, Oxford University) and inserted upstream of the RHO-1(G14V) cDNA to give pRJM098.

**C3 Transferase Transgenes.** The C3 Transferase coding sequence N-terminally tagged with GFP was as described previously (McMullan et al. 2006). The GFP tag was replaced with mCherry and C3Transferase::mCherry was inserted downstream of the *bus-1* promoter to give pRJM100. *nzEx492* contains an extrachromosomal version of this plasmid. The untagged C3 transferase coding sequence was inserted downstream of the 1.3Kb *egl-5* promoter fragment to give pRJM122. This was injected 1, 5 and 10ng/μl however we were unable to generate any transgenic lines that expressed C3 transferase under the control of this promoter.

**LET-60 Transgenes.** The wild-type LET-60 cDNA was obtained from yk1438c09 (a gift of Yuji Kohara). The sequence was modified using a Quickchange kit (Agilent technologies) to create the G12V mutation and subcloned into either the pPD49_78 heat shock vector (a gift of A. Fire Stanford University CA) (pRJM137) or a vector driving expression from the 1.3Kb *egl-5* promoter fragment (pRJM141). These plasmids were injected at 10ng/μl. *nzEx532* and *nzEx534* contain extrachromosomal versions of pRJM137 and pRJM141 respectively.

**MEK-2 Transgenes.** The MEK-2 cDNA was obtained from yk1736d7 (a gift of Yuji Kohara). The sequence was modified using Quickchange (Agilent technologies) to create the S223E and S227D mutations and subcloned into either the pPD49_78 heat shock vector (a gift of A. Fire Stanford University CA) (pRJM116) or a vector driving expression from the 1.3Kb *egl-5* promoter fragment (pRJM120). These plasmids were injected at 10ng/μl. *nzEx522* and *nzEx517* contain extrachromosomal versions of pRJM116 and pRJM120 respectively.

**UNC-73 Transgenes.** The 1.3Kb *egl-5* promoter fragment and *pes-10* minimal promoter was inserted upstream of an UNC-73D1 minigene tagged with GFP (Steven et al. 2005) (a gift of Rob Steven, University of Toledo, Ohio) (pRJM#095). *nzEx484* contains an extrachromosomal version of pRJM095 (injected at 20ng/μl) in an *unc-73(ce362)* background.

**EGL-30 (Gαq) Transgenes.** The wild type *egl-30* cDNA (Brundage et al. 1996) was subcloned into a vector driving expression from the 1.3Kb *egl-5* promoter fragment (pRJM156) or the *unc-17* promoter (pRJM160). pRJM156 was injected into *egl-30(ad805)* animals at 20ng/μl and *impEx005* contains an extrachromosomal version of pRJM156. pRJM160 was co-injected with SJN445 into *egl-30(ad805)* animals at 20ng/μl to generate QT1170 *nzEx584,*  only animals with motorneuron mcherry expression were used for assays*.* The wild type *egl-30* cdna sequence was modified using Quickchange to introduce the gain of function Q205L mutation and subcloned into either the pPD49_78 heat shock vector (a gift of A. Fire Stanford University CA) (pRJM155) or a vector driving expression from the 1.3Kb *egl-5* promoter fragment (pRJM158). These plasmids were injected at 1ng/μl. *impEx003* and *impEx004* contain extrachromosomal versions of pRJM155 and pRJM158 respectively.

**TABLE S1 additional *C. elegans* strains used**

| **Strain** | **Relevant Genotype** | **Source** | **Reference** |
| --- | --- | --- | --- |
| N2 Bristol | Wild type | CGC | (Brenner 1974) |
| RB541 | *exc-5(ok271)* | CGC |  |
| RB1094 | *C02F12.7(ok1072)* | CGC |  |
| VC356 | *C28C12.10(gk206)* | CGC |  |
| RB1521 | *Y95B8A.12(ok1820)* | CGC |  |
| RB907 | *C11D9.1(ok772)* | CGC |  |
| VC506 | *C14A11.3a(gk261)* | CGC |  |
| MH2407 | *ect-2(ku427)* | CGC | (Morita et al. 2005) |
| CB1460 | *unc-89(e1460)* | CGC |  |
| CB936 | *unc-73(e936)* | CGC | (Desai et al. 1988) |
| VC630 | *unc-73(ok936)* | CGC | (Steven et al. 2005) |
| DA823 | *egl-30(ad805)* | CGC | (Brundage et al. 1996) |
| NM1380 | *egl-30(js126gf)* | CGC | (Brundage et al. 1996) |
| PS427 | *lin-45(sy96)* | CGC | (Han et al. 1993) |
| MT8666 | *mek-2(n1989)* | CGC | (Kornfeld et al. 1995) |
| MH37 | *mpk-1(ku1);unc-32(e189)* | CGC | (Wu and Han 1994) |
| MT4866 | *let-60(n2021)* | CGC |  |
| PS436 | *let-60(sy93)* | CGC | (Han et al. 1990) |
| MT2124 | *let-60(n1046gf)* | CGC | (Ferguson and Horvitz 1985) |
| VC431 | *ras-1(gk237)* | CGC |  |
| RB852 | *ras-2(ok682)* | CGC |  |
| PJ1115 | *gaIs37*(EF1a::DMEK; hs::MPK-1) | CGC | (Berset et al. 2001) |
| UP1135 | *csEx52*(hs::LIN-45 S321A, S453A) | CGC | (Chong et al. 2001) |
| QT47 | *nzIs1*(hs::RHO-1*) |  | (McMullan et al. 2006) |
| QT230 | *rhgf-1(ok880)* |  | (Hiley et al. 2006) |
| KG1278 | *unc-73(ce362)* | Kenneth Miller (OMRF Oklahoma) | (Williams et al. 2007) |
| KG1358 | *ceEx195*(hs::UNC-73E);*unc-73(ce362)* | Kenneth Miller (OMRF Oklahoma) | (Williams et al. 2007) |
| EG317 | *unc-73(ox317)* | Kenneth Miller (OMRF Oklahoma) | (Williams et al. 2007) |
| GR1085 | *unc-29(e1072)* | Gary Ruvkun  (Harvard) |  |

**SUPPLEMENTAL FIGURES**

**Figure S1 EGL-30 (Gαq) signaling in the rectal epithelium fails to rescue severe constipation in infected *egl-30(ad805)* animals.** A. Uninfected *egl-30(ad805)* adult animals. An asterisk indicates the intestine. B. *egl-30(ad805)* animals infected with *M. nematophilum* are *bus* and severely constipated. C. Expression of EGL-30 (Gαq) in the rectal epithelial cells using a 1.3Kb *egl-5* promoter fragment rescues the *dar* phenotype following infection however these animals remain severely constipated. Extent of intestinal distention is indicated by double-headed arrows.

**Figure S2 Inhibition of the MAPK pathway suppresses the RHO-1* induced *dar*.** Adult wild-type animals and animals expressing hs::RHO-1* were pre-treated with 50μM of the MEK inhibitor U0126 (or DMSO as a control) for 2 hours at 20^o^C and then heat shocked as described in Material and Methods. After overnight recovery the percentage of animals showing the *dar* phenotype was scored. No *dar* response was observed in wild-type animals treated with either DMSO or U0126 (A and B). Animals expressing activated RHO-1* were *dar* (C) and this was blocked by pre-treatment with U0126 (C and D). Rectal opening is indicated with an arrow.

**SUPPLEMENTAL REFERENCES**

Berset, T., Hoier, E.F., Battu, G., Canevascini, S., and Hajnal, A. 2001. Notch inhibition of RAS signaling through MAP kinase phosphatase LIP-1 during C. elegans vulval development. *Science* **291**(5506): 1055-1058.

Brenner, S. 1974. The genetics of Caenorhabditis elegans. *Genetics* **77**(1): 71-94.

Brundage, L., Avery, L., Katz, A., Kim, U.J., Mendel, J.E., Sternberg, P.W., and Simon, M.I. 1996. Mutations in a C. elegans Gqalpha gene disrupt movement, egg laying, and viability. *Neuron* **16**(5): 999-1009.

Chong, H., Lee, J., and Guan, K.L. 2001. Positive and negative regulation of Raf kinase activity and function by phosphorylation. *EMBO J* **20**(14): 3716-3727.

Desai, C., Garriga, G., McIntire, S.L., and Horvitz, H.R. 1988. A genetic pathway for the development of the Caenorhabditis elegans HSN motor neurons. *Nature* **336**(6200): 638-646.

Ferguson, E.L. and Horvitz, H.R. 1985. Identification and characterization of 22 genes that affect the vulval cell lineages of the nematode Caenorhabditis elegans. *Genetics* **110**(1): 17-72.

Gravato-Nobre, M.J. and Hodgkin, J. 2008. The acyltransferase gene bus-1 exhibits conserved and specific expression in nematode rectal cells and reveals pathogen-induced cell swelling. *Dev Dyn* **237**(12): 3762-3776.

Han, M., Aroian, R.V., and Sternberg, P.W. 1990. The let-60 locus controls the switch between vulval and nonvulval cell fates in Caenorhabditis elegans. *Genetics* **126**(4): 899-913.

Han, M., Golden, A., Han, Y., and Sternberg, P.W. 1993. C. elegans lin-45 raf gene participates in let-60 ras-stimulated vulval differentiation. *Nature* **363**(6425): 133-140.

Hiley, E., McMullan, R., and Nurrish, S.J. 2006. The Galpha12-RGS RhoGEF-RhoA signalling pathway regulates neurotransmitter release in C. elegans. *EMBO J* **25**(24): 5884-5895.

Kornfeld, K., Guan, K.L., and Horvitz, H.R. 1995. The Caenorhabditis elegans gene mek-2 is required for vulval induction and encodes a protein similar to the protein kinase MEK. *Genes Dev* **9**(6): 756-768.

McMullan, R., Hiley, E., Morrison, P., and Nurrish, S.J. 2006. Rho is a presynaptic activator of neurotransmitter release at pre-existing synapses in C. elegans. *Genes Dev* **20**(1): 65-76.

Morita, K., Hirono, K., and Han, M. 2005. The Caenorhabditis elegans ect-2 RhoGEF gene regulates cytokinesis and migration of epidermal P cells. *EMBO Rep* **6**(12): 1163-1168.

Nicholas, H. and Hodgkin, J. 2009. The C. elegans Hox gene egl-5 is required for correct development of the hermaphrodite hindgut and for the response to rectal infection by Microbacterium nematophilum. *Dev Biol*.

Steven, R., Zhang, L., Culotti, J., and Pawson, T. 2005. The UNC-73/Trio RhoGEF-2 domain is required in separate isoforms for the regulation of pharynx pumping and normal neurotransmission in C. elegans. *Genes Dev* **19**(17): 2016-2029.

Teng, Y., Girard, L., Ferreira, H.B., Sternberg, P.W., and Emmons, S.W. 2004. Dissection of cis-regulatory elements in the C. elegans Hox gene egl-5 promoter. *Dev Biol* **276**(2): 476-492.

Williams, S.L., Lutz, S., Charlie, N.K., Vettel, C., Ailion, M., Coco, C., Tesmer, J.J., Jorgensen, E.M., Wieland, T., and Miller, K.G. 2007. Trio's Rho-specific GEF domain is the missing Galpha q effector in C. elegans. *Genes Dev* **21**(21): 2731-2746.

Wu, Y. and Han, M. 1994. Suppression of activated Let-60 ras protein defines a role of Caenorhabditis elegans Sur-1 MAP kinase in vulval differentiation. *Genes Dev* **8**(2): 147-159.
